# 1,3-Dibenzyl-5-chloro-1*H*-benzimidazol-2(3*H*)-one

**DOI:** 10.1107/S1600536811029084

**Published:** 2011-07-23

**Authors:** Rachida Dardouri, Youssef Kandri Rodi, Sonia Ladeira, El Mokhtar Essassi, Seik Weng Ng

**Affiliations:** a Laboratoire de Chimie Organique Appliquée, Faculté des Sciences et Techniques Université Sidi Mohamed Ben Abdallah, Fés, Morocco; b Service Commun Rayons-X FR2599, Université Paul Sabatier, Bâtiment 2R1, 118 route de Narbonne, Toulouse, France; c Laboratoire de Chimie Organique Hétérocyclique, Pôle de Compétences Pharmacochimie, Université Mohammed V-Agdal, BP 1014 Avenue Ibn Batout, Rabat, Morocco; d Department of Chemistry, University of Malaya, 50603 Kuala Lumpur, Malaysia; e Chemistry Department, Faculty of Science, King Abdulaziz University, PO Box 80203 Jeddah, Saudi Arabia

## Abstract

In both independent mol­ecules of the title compound, C_21_H_17_ClN_2_O, the aromatic rings of the benzyl substituents are located on opposite sides of the benzimidazole  ring systems. In one mol­ecule, the rings are aligned at 77.0 (1) and 78.1 (1)° with respect to the fused-ring system, whereas in the other mol­ecule the rings are aligned at 76.0 (1) and 76.9 (1)°. There is an inter­molecular Cl⋯O contact of 3.086 (1) Å.

## Related literature

For the structure of monobenzyl-benzimidazol-3-one, see: Ouzidan *et al.* (2011 ▶).
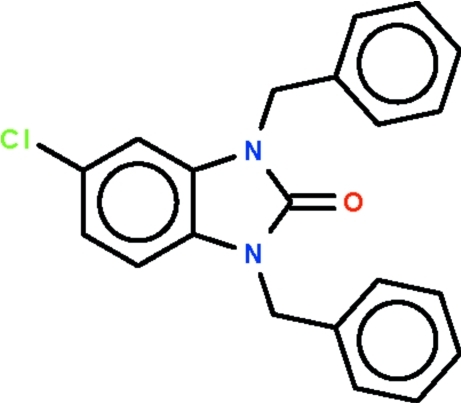


## Experimental

### 

#### Crystal data

C_21_H_17_ClN_2_O
*M* *_r_* = 348.82
Monoclinic, 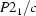

*a* = 11.0380 (4) Å
*b* = 9.2863 (3) Å
*c* = 33.2679 (13) Å
β = 92.297 (2)°
*V* = 3407.3 (2) Å^3^
*Z* = 8
Mo *K*α radiation
μ = 0.24 mm^−1^
*T* = 293 K
0.08 × 0.04 × 0.03 mm

#### Data collection

Bruker X8 APEXII diffractometer
Absorption correction: multi-scan (*SADABS*; Sheldrick, 1996 ▶) *T*_min_ = 0.981, *T*_max_ = 0.993
22403 measured reflections
5950 independent reflections
3942 reflections with *I* > 2σ(*I*)
*R*_int_ = 0.073

#### Refinement

*R*[*F* ^2^ > 2σ(*F* ^2^)] = 0.058
*wR*(*F* ^2^) = 0.159
*S* = 1.03
5950 reflections
451 parameters
H-atom parameters constrained
Δρ_max_ = 0.77 e Å^−3^
Δρ_min_ = −0.36 e Å^−3^

### 

Data collection: *APEX2* (Bruker, 2008 ▶); cell refinement: *SAINT* (Bruker, 2008 ▶); data reduction: *SAINT*; program(s) used to solve structure: *SHELXS97* (Sheldrick, 2008 ▶); program(s) used to refine structure: *SHELXL97* (Sheldrick, 2008 ▶); molecular graphics: *X-SEED* (Barbour, 2001 ▶); software used to prepare material for publication: *publCIF* (Westrip, 2010 ▶).

## Supplementary Material

Crystal structure: contains datablock(s) global, I. DOI: 10.1107/S1600536811029084/bt6817sup1.cif

Structure factors: contains datablock(s) I. DOI: 10.1107/S1600536811029084/bt6817Isup2.hkl

Supplementary material file. DOI: 10.1107/S1600536811029084/bt6817Isup3.cml

Additional supplementary materials:  crystallographic information; 3D view; checkCIF report

## Figures and Tables

**Fig. 1.**
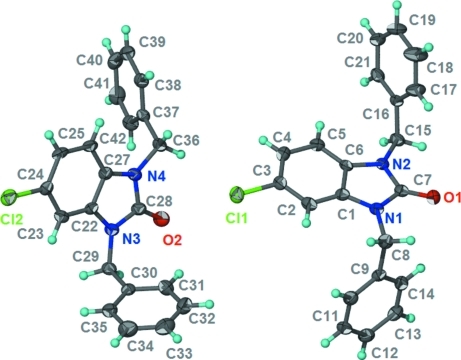
Anisotropic displacement ellipsoid plot (Barbour, 2001) of the two molecules of C21H17ClN2O at the 70% probability level; hydrogen atoms are drawn as spheres of an arbitrary radius.

## supplementary crystallographic information

### Comment

A recent study reported the synthesis of monobenzyl-benzimidazol-3-one by the reaction of benzyl chloride on benzimidazol-3-one (Ouzidan *et al.*, 2011). The use of double the molar quantity of benzyl choride on 5-chlorobenzimidazol-3-one yielded the expected title dibenzyl analog (Scheme I, Fig. 1). In both independent molecules, the aromatic rings of the benzyl substituent lie of opposite sides of the planar benzimidazole fused-ring. In one molecule, the rings are aligned at 77.0 (1)° and 78.1 (1)° with respect to the fused-ring whereas in the other, the rings are aligned at 76.0 (1)° and 76.9 (1)°.

### Experimental

To 5-chloro-1*H*-benzimidazol-2(3*H*)-one (0.2 g, 1.18 mmol), potassium carbonate (0.40 g, 2.80 mmol), and tetra-*n*-butylammonium bromide (0.08 g, 0.23 mmol) in DMF (15 ml) was added benzyl chloride (0.33 g, 2.6 mmol). Stirring was continued at room temperature for 6 h. The salts were removed by filtration and the filtrate concentrated under reduced pressure. The residue was separated by chromatography on a column of silica gel with ethyl acetate/hexane (1/2) as eluent. The compound was recrystallized from hexane.

### Refinement

Carbon-bound H-atoms were placed in calculated positions (C—H 0.93–0.97 Å) and were included in the refinement in the riding model approximation, with *U*(H) set to 1.2*U*_eq_(C).

Omitted from the refinement were (-1 0 2), (1 0 2), (0 1 2), (0 0 2), (0 1 1) and (-1 1 2) owing to bad agreement between observed and calculated structure factors.

### Crystal data

**Table:**

C_21_H_17_ClN_2_O	*F*(000) = 1456
*M**_r_* = 348.82	*D*_x_ = 1.360 Mg m^−^^3^
Monoclinic, *P*2_1_/*c*	Mo *K*α radiation, λ = 0.71073 Å
Hall symbol: -P 2ybc	Cell parameters from 2455 reflections
*a* = 11.0380 (4) Å	θ = 2.5–24.4°
*b* = 9.2863 (3) Å	µ = 0.24 mm^−^^1^
*c* = 33.2679 (13) Å	*T* = 293 K
β = 92.297 (2)°	Prism, colorless
*V* = 3407.3 (2) Å^3^	0.08 × 0.04 × 0.03 mm
*Z* = 8	

### Data collection

**Table:**

Bruker X8 APEXII diffractometer	5950 independent reflections
Radiation source: fine-focus sealed tube	3942 reflections with *I* > 2σ(*I*)
graphite	*R*_int_ = 0.073
φ and ω scans	θ_max_ = 25.0°, θ_min_ = 1.9°
Absorption correction: multi-scan (*SADABS*; Sheldrick, 1996)	*h* = −13→13
*T*_min_ = 0.981, *T*_max_ = 0.993	*k* = −11→10
22403 measured reflections	*l* = −39→39

### Refinement

**Table:**

Refinement on *F*^2^	Primary atom site location: structure-invariant direct methods
Least-squares matrix: full	Secondary atom site location: difference Fourier map
*R*[*F*^2^ > 2σ(*F*^2^)] = 0.058	Hydrogen site location: inferred from neighbouring sites
*wR*(*F*^2^) = 0.159	H-atom parameters constrained
*S* = 1.03	*w* = 1/[σ^2^(*F*_o_^2^) + (0.071*P*)^2^ + 2.3735*P*] where *P* = (*F*_o_^2^ + 2*F*_c_^2^)/3
5950 reflections	(Δ/σ)_max_ = 0.001
451 parameters	Δρ_max_ = 0.77 e Å^−^^3^
0 restraints	Δρ_min_ = −0.36 e Å^−^^3^

### Fractional atomic coordinates and isotropic or equivalent isotropic displacement parameters (Å^2^)

**Table:**

	*x*	*y*	*z*	*U*_iso_*/*U*_eq_	
Cl1	0.34242 (8)	0.76198 (10)	0.10621 (3)	0.0370 (3)	
Cl2	−0.16981 (8)	1.64706 (10)	0.03835 (3)	0.0392 (3)	
O1	0.6486 (2)	0.2235 (3)	0.23982 (7)	0.0360 (6)	
O2	0.1395 (2)	0.9431 (3)	0.06445 (8)	0.0391 (7)	
N1	0.5083 (2)	0.3850 (3)	0.21135 (8)	0.0290 (7)	
N2	0.6912 (2)	0.3771 (3)	0.18714 (9)	0.0286 (7)	
N3	−0.0049 (2)	1.1228 (3)	0.05254 (9)	0.0287 (7)	
N4	0.1826 (2)	1.1884 (3)	0.06987 (9)	0.0299 (7)	
C1	0.5083 (3)	0.4824 (4)	0.17969 (10)	0.0277 (8)	
C2	0.4193 (3)	0.5703 (4)	0.16282 (10)	0.0297 (8)	
H2	0.3415	0.5730	0.1725	0.036*	
C3	0.4529 (3)	0.6548 (4)	0.13040 (10)	0.0282 (8)	
C4	0.5684 (3)	0.6555 (4)	0.11589 (11)	0.0332 (9)	
H4	0.5868	0.7160	0.0947	0.040*	
C5	0.6573 (3)	0.5652 (4)	0.13307 (11)	0.0324 (9)	
H5	0.7353	0.5636	0.1235	0.039*	
C6	0.6258 (3)	0.4786 (4)	0.16458 (10)	0.0256 (8)	
C7	0.6200 (3)	0.3167 (4)	0.21566 (10)	0.0288 (8)	
C8	0.4026 (3)	0.3441 (4)	0.23430 (10)	0.0325 (9)	
H8A	0.3534	0.4287	0.2387	0.039*	
H8B	0.4300	0.3070	0.2604	0.039*	
C9	0.3263 (3)	0.2313 (4)	0.21252 (10)	0.0264 (8)	
C10	0.2142 (3)	0.2636 (4)	0.19518 (11)	0.0320 (9)	
H10	0.1824	0.3557	0.1977	0.038*	
C11	0.1489 (3)	0.1595 (4)	0.17398 (11)	0.0350 (9)	
H11	0.0736	0.1825	0.1622	0.042*	
C12	0.1940 (3)	0.0227 (4)	0.17016 (11)	0.0364 (9)	
H12	0.1501	−0.0465	0.1556	0.044*	
C13	0.3058 (3)	−0.0117 (4)	0.18826 (11)	0.0355 (9)	
H13	0.3364	−0.1047	0.1863	0.043*	
C14	0.3710 (3)	0.0919 (4)	0.20903 (10)	0.0326 (9)	
H14	0.4461	0.0687	0.2210	0.039*	
C15	0.8138 (3)	0.3320 (4)	0.17933 (11)	0.0316 (9)	
H15A	0.8174	0.3031	0.1514	0.038*	
H15B	0.8342	0.2487	0.1959	0.038*	
C16	0.9066 (3)	0.4487 (4)	0.18787 (10)	0.0254 (8)	
C17	0.9235 (3)	0.5040 (5)	0.22652 (11)	0.0429 (10)	
H17	0.8761	0.4707	0.2471	0.051*	
C18	1.0100 (4)	0.6077 (5)	0.23439 (13)	0.0555 (13)	
H18	1.0211	0.6436	0.2604	0.067*	
C19	1.0807 (3)	0.6594 (4)	0.20421 (12)	0.0419 (10)	
H19	1.1393	0.7292	0.2098	0.050*	
C20	1.0637 (3)	0.6071 (4)	0.16622 (11)	0.0359 (9)	
H20	1.1104	0.6420	0.1457	0.043*	
C21	0.9772 (3)	0.5022 (4)	0.15803 (11)	0.0320 (9)	
H21	0.9664	0.4671	0.1319	0.038*	
C22	−0.0032 (3)	1.2731 (4)	0.05389 (10)	0.0252 (8)	
C23	−0.0928 (3)	1.3738 (4)	0.04580 (10)	0.0285 (8)	
H23	−0.1721	1.3468	0.0390	0.034*	
C24	−0.0587 (3)	1.5167 (4)	0.04828 (10)	0.0269 (8)	
C25	0.0588 (3)	1.5596 (4)	0.05836 (10)	0.0307 (8)	
H25	0.0784	1.6570	0.0595	0.037*	
C26	0.1466 (3)	1.4569 (4)	0.06670 (10)	0.0301 (8)	
H26	0.2257	1.4841	0.0738	0.036*	
C27	0.1156 (3)	1.3146 (4)	0.06442 (10)	0.0268 (8)	
C28	0.1094 (3)	1.0696 (4)	0.06260 (10)	0.0299 (8)	
C29	−0.1116 (3)	1.0311 (4)	0.04639 (10)	0.0301 (8)	
H29A	−0.0863	0.9373	0.0370	0.036*	
H29B	−0.1649	1.0730	0.0257	0.036*	
C30	−0.1805 (3)	1.0127 (4)	0.08438 (10)	0.0280 (8)	
C31	−0.1280 (3)	0.9383 (4)	0.11718 (12)	0.0390 (10)	
H31	−0.0511	0.8989	0.1152	0.047*	
C32	−0.1877 (4)	0.9221 (4)	0.15238 (12)	0.0450 (10)	
H32	−0.1513	0.8723	0.1739	0.054*	
C33	−0.3026 (4)	0.9806 (5)	0.15555 (13)	0.0489 (11)	
H33	−0.3434	0.9706	0.1793	0.059*	
C34	−0.3563 (4)	1.0533 (5)	0.12353 (13)	0.0475 (11)	
H34	−0.4336	1.0918	0.1256	0.057*	
C35	−0.2953 (3)	1.0695 (4)	0.08817 (12)	0.0371 (9)	
H35	−0.3321	1.1192	0.0667	0.045*	
C36	0.3085 (3)	1.1791 (4)	0.08465 (11)	0.0316 (9)	
H36A	0.3262	1.0804	0.0924	0.038*	
H36B	0.3192	1.2386	0.1085	0.038*	
C37	0.3976 (3)	1.2263 (4)	0.05408 (10)	0.0275 (8)	
C38	0.4760 (3)	1.3383 (4)	0.06241 (11)	0.0314 (9)	
H38	0.4722	1.3870	0.0868	0.038*	
C39	0.5609 (3)	1.3799 (4)	0.03490 (12)	0.0387 (10)	
H39	0.6130	1.4566	0.0407	0.046*	
C40	0.5674 (4)	1.3065 (4)	−0.00092 (12)	0.0411 (10)	
H40	0.6247	1.3327	−0.0193	0.049*	
C41	0.4894 (4)	1.1950 (5)	−0.00948 (12)	0.0416 (10)	
H41	0.4943	1.1458	−0.0337	0.050*	
C42	0.4036 (3)	1.1548 (4)	0.01745 (11)	0.0352 (9)	
H42	0.3500	1.0801	0.0111	0.042*	

### Atomic displacement parameters (Å^2^)

**Table:**

	*U*^11^	*U*^22^	*U*^33^	*U*^12^	*U*^13^	*U*^23^
Cl1	0.0347 (5)	0.0362 (6)	0.0402 (5)	0.0082 (4)	0.0016 (4)	0.0039 (4)
Cl2	0.0399 (5)	0.0284 (6)	0.0497 (6)	0.0047 (4)	0.0066 (4)	0.0039 (4)
O1	0.0320 (14)	0.0387 (16)	0.0369 (14)	−0.0017 (12)	−0.0031 (11)	0.0085 (12)
O2	0.0315 (14)	0.0280 (16)	0.0577 (18)	0.0055 (12)	0.0008 (12)	0.0016 (13)
N1	0.0250 (15)	0.0309 (18)	0.0312 (16)	−0.0041 (13)	0.0011 (12)	0.0009 (13)
N2	0.0198 (14)	0.0301 (18)	0.0356 (17)	−0.0008 (13)	−0.0011 (12)	0.0049 (13)
N3	0.0226 (15)	0.0261 (18)	0.0374 (17)	−0.0011 (13)	0.0002 (12)	−0.0005 (13)
N4	0.0205 (15)	0.0319 (19)	0.0373 (17)	0.0009 (13)	0.0014 (12)	0.0021 (13)
C1	0.0258 (18)	0.025 (2)	0.0323 (19)	−0.0032 (16)	0.0009 (15)	−0.0028 (16)
C2	0.0228 (18)	0.030 (2)	0.036 (2)	0.0030 (16)	0.0009 (15)	−0.0078 (17)
C3	0.0293 (19)	0.019 (2)	0.036 (2)	0.0039 (15)	−0.0029 (15)	−0.0025 (16)
C4	0.034 (2)	0.032 (2)	0.034 (2)	−0.0004 (17)	0.0013 (16)	0.0058 (17)
C5	0.0253 (19)	0.035 (2)	0.037 (2)	−0.0041 (17)	0.0012 (15)	0.0005 (17)
C6	0.0199 (17)	0.026 (2)	0.0308 (19)	−0.0032 (15)	−0.0039 (14)	−0.0029 (15)
C7	0.028 (2)	0.029 (2)	0.0294 (19)	−0.0061 (16)	−0.0041 (15)	−0.0024 (17)
C8	0.032 (2)	0.038 (2)	0.0282 (19)	−0.0009 (17)	0.0045 (15)	−0.0019 (17)
C9	0.0237 (18)	0.028 (2)	0.0284 (19)	−0.0016 (15)	0.0091 (14)	0.0002 (15)
C10	0.0276 (19)	0.031 (2)	0.038 (2)	0.0022 (17)	0.0055 (16)	0.0024 (17)
C11	0.0220 (18)	0.044 (3)	0.039 (2)	−0.0030 (17)	0.0007 (16)	0.0032 (18)
C12	0.039 (2)	0.035 (2)	0.035 (2)	−0.0101 (18)	0.0020 (17)	−0.0018 (17)
C13	0.045 (2)	0.025 (2)	0.037 (2)	−0.0007 (18)	0.0072 (18)	0.0018 (17)
C14	0.0296 (19)	0.037 (2)	0.031 (2)	0.0005 (17)	0.0014 (15)	0.0065 (17)
C15	0.0218 (18)	0.033 (2)	0.039 (2)	0.0016 (16)	−0.0010 (15)	0.0000 (17)
C16	0.0170 (16)	0.029 (2)	0.0297 (18)	−0.0004 (15)	0.0006 (14)	−0.0001 (15)
C17	0.038 (2)	0.060 (3)	0.031 (2)	−0.021 (2)	0.0109 (17)	−0.0048 (19)
C18	0.050 (3)	0.077 (3)	0.040 (2)	−0.029 (2)	0.008 (2)	−0.024 (2)
C19	0.032 (2)	0.042 (3)	0.052 (3)	−0.0153 (19)	0.0048 (18)	−0.008 (2)
C20	0.0266 (19)	0.040 (2)	0.042 (2)	−0.0051 (18)	0.0062 (16)	0.0070 (18)
C21	0.0224 (18)	0.044 (2)	0.0298 (19)	0.0029 (17)	−0.0001 (15)	0.0023 (17)
C22	0.0250 (18)	0.025 (2)	0.0261 (18)	−0.0022 (16)	0.0072 (14)	0.0016 (15)
C23	0.0221 (18)	0.034 (2)	0.0293 (19)	−0.0020 (16)	0.0018 (14)	−0.0025 (16)
C24	0.0302 (19)	0.028 (2)	0.0230 (17)	0.0006 (16)	0.0089 (14)	0.0019 (15)
C25	0.035 (2)	0.027 (2)	0.031 (2)	−0.0110 (17)	0.0089 (16)	0.0000 (16)
C26	0.0259 (18)	0.033 (2)	0.032 (2)	−0.0083 (17)	0.0056 (15)	0.0019 (16)
C27	0.0250 (18)	0.028 (2)	0.0274 (18)	0.0008 (16)	0.0063 (14)	0.0004 (15)
C28	0.0233 (18)	0.034 (2)	0.033 (2)	−0.0017 (17)	0.0062 (15)	0.0008 (17)
C29	0.0276 (19)	0.025 (2)	0.037 (2)	−0.0024 (16)	−0.0019 (15)	−0.0032 (16)
C30	0.0288 (19)	0.020 (2)	0.035 (2)	−0.0052 (15)	−0.0018 (15)	−0.0013 (15)
C31	0.030 (2)	0.041 (3)	0.046 (2)	0.0028 (18)	−0.0019 (17)	0.0043 (19)
C32	0.051 (3)	0.043 (3)	0.040 (2)	−0.006 (2)	−0.0014 (19)	0.0093 (19)
C33	0.055 (3)	0.049 (3)	0.044 (2)	−0.010 (2)	0.014 (2)	−0.001 (2)
C34	0.034 (2)	0.048 (3)	0.061 (3)	0.003 (2)	0.014 (2)	0.001 (2)
C35	0.030 (2)	0.035 (2)	0.047 (2)	−0.0013 (17)	0.0017 (17)	0.0068 (18)
C36	0.0208 (18)	0.040 (2)	0.034 (2)	0.0010 (16)	0.0002 (15)	0.0036 (17)
C37	0.0177 (17)	0.029 (2)	0.036 (2)	0.0032 (15)	0.0010 (14)	0.0019 (16)
C38	0.0260 (19)	0.032 (2)	0.037 (2)	0.0043 (17)	0.0013 (15)	−0.0012 (17)
C39	0.027 (2)	0.028 (2)	0.060 (3)	0.0050 (17)	0.0038 (18)	0.0095 (19)
C40	0.038 (2)	0.043 (3)	0.044 (2)	0.008 (2)	0.0166 (18)	0.013 (2)
C41	0.045 (2)	0.046 (3)	0.034 (2)	0.010 (2)	0.0107 (18)	−0.0003 (18)
C42	0.031 (2)	0.031 (2)	0.043 (2)	0.0032 (17)	−0.0011 (17)	−0.0048 (18)

### Geometric parameters (Å, °)

**Table:**

Cl1—C3	1.745 (3)	C17—H17	0.9300
Cl2—C24	1.745 (3)	C18—C19	1.382 (6)
O1—C7	1.214 (4)	C18—H18	0.9300
O2—C28	1.222 (4)	C19—C20	1.360 (5)
N1—C7	1.389 (4)	C19—H19	0.9300
N1—C1	1.389 (4)	C20—C21	1.384 (5)
N1—C8	1.470 (4)	C20—H20	0.9300
N2—C7	1.376 (4)	C21—H21	0.9300
N2—C6	1.389 (4)	C22—C23	1.380 (5)
N2—C15	1.450 (4)	C22—C27	1.397 (5)
N3—C28	1.384 (4)	C23—C24	1.380 (5)
N3—C22	1.396 (4)	C23—H23	0.9300
N3—C29	1.461 (4)	C24—C25	1.386 (5)
N4—C28	1.383 (5)	C25—C26	1.380 (5)
N4—C27	1.393 (4)	C25—H25	0.9300
N4—C36	1.459 (4)	C26—C27	1.367 (5)
C1—C2	1.379 (5)	C26—H26	0.9300
C1—C6	1.410 (5)	C29—C30	1.511 (5)
C2—C3	1.396 (5)	C29—H29A	0.9700
C2—H2	0.9300	C29—H29B	0.9700
C3—C4	1.381 (5)	C30—C35	1.383 (5)
C4—C5	1.396 (5)	C30—C31	1.398 (5)
C4—H4	0.9300	C31—C32	1.375 (5)
C5—C6	1.377 (5)	C31—H31	0.9300
C5—H5	0.9300	C32—C33	1.388 (6)
C8—C9	1.511 (5)	C32—H32	0.9300
C8—H8A	0.9700	C33—C34	1.375 (6)
C8—H8B	0.9700	C33—H33	0.9300
C9—C10	1.377 (5)	C34—C35	1.387 (5)
C9—C14	1.392 (5)	C34—H34	0.9300
C10—C11	1.382 (5)	C35—H35	0.9300
C10—H10	0.9300	C36—C37	1.508 (5)
C11—C12	1.373 (5)	C36—H36A	0.9700
C11—H11	0.9300	C36—H36B	0.9700
C12—C13	1.388 (5)	C37—C38	1.375 (5)
C12—H12	0.9300	C37—C42	1.392 (5)
C13—C14	1.372 (5)	C38—C39	1.391 (5)
C13—H13	0.9300	C38—H38	0.9300
C14—H14	0.9300	C39—C40	1.377 (6)
C15—C16	1.510 (5)	C39—H39	0.9300
C15—H15A	0.9700	C40—C41	1.369 (6)
C15—H15B	0.9700	C40—H40	0.9300
C16—C21	1.379 (5)	C41—C42	1.381 (5)
C16—C17	1.390 (5)	C41—H41	0.9300
C17—C18	1.374 (5)	C42—H42	0.9300
			
C7—N1—C1	110.4 (3)	C19—C20—C21	120.2 (3)
C7—N1—C8	123.3 (3)	C19—C20—H20	119.9
C1—N1—C8	125.9 (3)	C21—C20—H20	119.9
C7—N2—C6	110.5 (3)	C16—C21—C20	121.1 (3)
C7—N2—C15	124.7 (3)	C16—C21—H21	119.4
C6—N2—C15	124.6 (3)	C20—C21—H21	119.4
C28—N3—C22	109.7 (3)	C23—C22—N3	131.5 (3)
C28—N3—C29	123.2 (3)	C23—C22—C27	121.3 (3)
C22—N3—C29	126.7 (3)	N3—C22—C27	107.2 (3)
C28—N4—C27	110.2 (3)	C22—C23—C24	116.6 (3)
C28—N4—C36	123.6 (3)	C22—C23—H23	121.7
C27—N4—C36	126.0 (3)	C24—C23—H23	121.7
C2—C1—N1	132.2 (3)	C23—C24—C25	122.8 (3)
C2—C1—C6	121.3 (3)	C23—C24—Cl2	117.9 (3)
N1—C1—C6	106.4 (3)	C25—C24—Cl2	119.3 (3)
C1—C2—C3	116.1 (3)	C26—C25—C24	119.5 (3)
C1—C2—H2	121.9	C26—C25—H25	120.2
C3—C2—H2	121.9	C24—C25—H25	120.2
C4—C3—C2	123.4 (3)	C27—C26—C25	119.0 (3)
C4—C3—Cl1	118.3 (3)	C27—C26—H26	120.5
C2—C3—Cl1	118.4 (3)	C25—C26—H26	120.5
C3—C4—C5	119.9 (3)	C26—C27—N4	132.5 (3)
C3—C4—H4	120.1	C26—C27—C22	120.7 (3)
C5—C4—H4	120.1	N4—C27—C22	106.8 (3)
C6—C5—C4	117.9 (3)	O2—C28—N4	127.1 (3)
C6—C5—H5	121.1	O2—C28—N3	126.8 (3)
C4—C5—H5	121.1	N4—C28—N3	106.1 (3)
C5—C6—N2	131.8 (3)	N3—C29—C30	112.3 (3)
C5—C6—C1	121.4 (3)	N3—C29—H29A	109.2
N2—C6—C1	106.9 (3)	C30—C29—H29A	109.2
O1—C7—N2	127.4 (3)	N3—C29—H29B	109.2
O1—C7—N1	126.8 (3)	C30—C29—H29B	109.2
N2—C7—N1	105.8 (3)	H29A—C29—H29B	107.9
N1—C8—C9	111.6 (3)	C35—C30—C31	118.0 (3)
N1—C8—H8A	109.3	C35—C30—C29	121.8 (3)
C9—C8—H8A	109.3	C31—C30—C29	120.2 (3)
N1—C8—H8B	109.3	C32—C31—C30	121.4 (4)
C9—C8—H8B	109.3	C32—C31—H31	119.3
H8A—C8—H8B	108.0	C30—C31—H31	119.3
C10—C9—C14	118.9 (3)	C31—C32—C33	119.5 (4)
C10—C9—C8	121.6 (3)	C31—C32—H32	120.2
C14—C9—C8	119.5 (3)	C33—C32—H32	120.2
C9—C10—C11	120.2 (3)	C34—C33—C32	120.0 (4)
C9—C10—H10	119.9	C34—C33—H33	120.0
C11—C10—H10	119.9	C32—C33—H33	120.0
C12—C11—C10	120.7 (3)	C33—C34—C35	120.1 (4)
C12—C11—H11	119.6	C33—C34—H34	119.9
C10—C11—H11	119.6	C35—C34—H34	119.9
C11—C12—C13	119.5 (4)	C30—C35—C34	120.9 (4)
C11—C12—H12	120.3	C30—C35—H35	119.5
C13—C12—H12	120.3	C34—C35—H35	119.5
C14—C13—C12	119.7 (4)	N4—C36—C37	113.2 (3)
C14—C13—H13	120.1	N4—C36—H36A	108.9
C12—C13—H13	120.1	C37—C36—H36A	108.9
C13—C14—C9	120.9 (3)	N4—C36—H36B	108.9
C13—C14—H14	119.5	C37—C36—H36B	108.9
C9—C14—H14	119.5	H36A—C36—H36B	107.8
N2—C15—C16	112.9 (3)	C38—C37—C42	119.1 (3)
N2—C15—H15A	109.0	C38—C37—C36	120.4 (3)
C16—C15—H15A	109.0	C42—C37—C36	120.5 (3)
N2—C15—H15B	109.0	C37—C38—C39	120.8 (3)
C16—C15—H15B	109.0	C37—C38—H38	119.6
H15A—C15—H15B	107.8	C39—C38—H38	119.6
C21—C16—C17	118.3 (3)	C40—C39—C38	119.5 (4)
C21—C16—C15	121.4 (3)	C40—C39—H39	120.3
C17—C16—C15	120.2 (3)	C38—C39—H39	120.3
C18—C17—C16	120.1 (3)	C41—C40—C39	120.0 (4)
C18—C17—H17	120.0	C41—C40—H40	120.0
C16—C17—H17	120.0	C39—C40—H40	120.0
C17—C18—C19	120.9 (4)	C40—C41—C42	120.8 (4)
C17—C18—H18	119.6	C40—C41—H41	119.6
C19—C18—H18	119.6	C42—C41—H41	119.6
C20—C19—C18	119.4 (4)	C41—C42—C37	119.8 (4)
C20—C19—H19	120.3	C41—C42—H42	120.1
C18—C19—H19	120.3	C37—C42—H42	120.1
			
C7—N1—C1—C2	177.0 (4)	C28—N3—C22—C23	178.8 (3)
C8—N1—C1—C2	4.2 (6)	C29—N3—C22—C23	−8.1 (6)
C7—N1—C1—C6	−2.1 (4)	C28—N3—C22—C27	1.0 (4)
C8—N1—C1—C6	−174.9 (3)	C29—N3—C22—C27	174.0 (3)
N1—C1—C2—C3	−179.5 (3)	N3—C22—C23—C24	−177.0 (3)
C6—C1—C2—C3	−0.4 (5)	C27—C22—C23—C24	0.6 (5)
C1—C2—C3—C4	−1.4 (5)	C22—C23—C24—C25	−0.1 (5)
C1—C2—C3—Cl1	177.0 (3)	C22—C23—C24—Cl2	−179.8 (2)
C2—C3—C4—C5	2.0 (6)	C23—C24—C25—C26	−0.6 (5)
Cl1—C3—C4—C5	−176.5 (3)	Cl2—C24—C25—C26	179.2 (3)
C3—C4—C5—C6	−0.6 (5)	C24—C25—C26—C27	0.7 (5)
C4—C5—C6—N2	178.6 (3)	C25—C26—C27—N4	178.0 (3)
C4—C5—C6—C1	−1.2 (5)	C25—C26—C27—C22	−0.1 (5)
C7—N2—C6—C5	−179.6 (4)	C28—N4—C27—C26	−177.6 (4)
C15—N2—C6—C5	−4.8 (6)	C36—N4—C27—C26	7.7 (6)
C7—N2—C6—C1	0.2 (4)	C28—N4—C27—C22	0.7 (4)
C15—N2—C6—C1	175.0 (3)	C36—N4—C27—C22	−174.0 (3)
C2—C1—C6—C5	1.7 (5)	C23—C22—C27—C26	−0.5 (5)
N1—C1—C6—C5	−179.0 (3)	N3—C22—C27—C26	177.6 (3)
C2—C1—C6—N2	−178.1 (3)	C23—C22—C27—N4	−179.1 (3)
N1—C1—C6—N2	1.2 (4)	N3—C22—C27—N4	−1.0 (4)
C6—N2—C7—O1	178.9 (3)	C27—N4—C28—O2	179.6 (3)
C15—N2—C7—O1	4.0 (6)	C36—N4—C28—O2	−5.6 (6)
C6—N2—C7—N1	−1.5 (4)	C27—N4—C28—N3	−0.1 (4)
C15—N2—C7—N1	−176.3 (3)	C36—N4—C28—N3	174.7 (3)
C1—N1—C7—O1	−178.1 (3)	C22—N3—C28—O2	179.8 (3)
C8—N1—C7—O1	−5.1 (5)	C29—N3—C28—O2	6.4 (5)
C1—N1—C7—N2	2.2 (4)	C22—N3—C28—N4	−0.5 (4)
C8—N1—C7—N2	175.3 (3)	C29—N3—C28—N4	−173.9 (3)
C7—N1—C8—C9	−90.5 (4)	C28—N3—C29—C30	92.6 (4)
C1—N1—C8—C9	81.5 (4)	C22—N3—C29—C30	−79.6 (4)
N1—C8—C9—C10	−108.5 (4)	N3—C29—C30—C35	112.5 (4)
N1—C8—C9—C14	70.1 (4)	N3—C29—C30—C31	−66.6 (4)
C14—C9—C10—C11	−1.4 (5)	C35—C30—C31—C32	−0.3 (6)
C8—C9—C10—C11	177.2 (3)	C29—C30—C31—C32	178.8 (3)
C9—C10—C11—C12	0.5 (5)	C30—C31—C32—C33	0.1 (6)
C10—C11—C12—C13	0.8 (5)	C31—C32—C33—C34	0.4 (6)
C11—C12—C13—C14	−1.3 (5)	C32—C33—C34—C35	−0.6 (6)
C12—C13—C14—C9	0.5 (5)	C31—C30—C35—C34	0.2 (5)
C10—C9—C14—C13	0.9 (5)	C29—C30—C35—C34	−179.0 (3)
C8—C9—C14—C13	−177.8 (3)	C33—C34—C35—C30	0.3 (6)
C7—N2—C15—C16	−116.9 (4)	C28—N4—C36—C37	112.1 (4)
C6—N2—C15—C16	68.9 (4)	C27—N4—C36—C37	−73.9 (4)
N2—C15—C16—C21	−118.9 (4)	N4—C36—C37—C38	120.9 (4)
N2—C15—C16—C17	61.7 (4)	N4—C36—C37—C42	−60.6 (4)
C21—C16—C17—C18	−1.0 (6)	C42—C37—C38—C39	−0.4 (5)
C15—C16—C17—C18	178.4 (4)	C36—C37—C38—C39	178.1 (3)
C16—C17—C18—C19	0.5 (7)	C37—C38—C39—C40	−0.8 (5)
C17—C18—C19—C20	0.3 (7)	C38—C39—C40—C41	0.9 (6)
C18—C19—C20—C21	−0.6 (6)	C39—C40—C41—C42	0.1 (6)
C17—C16—C21—C20	0.7 (5)	C40—C41—C42—C37	−1.3 (6)
C15—C16—C21—C20	−178.7 (3)	C38—C37—C42—C41	1.4 (5)
C19—C20—C21—C16	0.1 (6)	C36—C37—C42—C41	−177.0 (3)
